# Context dependent cognitive development in Bhutanese children

**DOI:** 10.1038/s41598-023-47254-x

**Published:** 2023-11-14

**Authors:** Gustaf Gredebäck, Nidup Dorji, Umay Sen, Pär Nyström, Johanna Hellberg, Wangchuk

**Affiliations:** 1 Uppsala University, Uppsala, Sweden; 2 Khesar Gyalpo University of Medical Sciences of Bhutan, Thimphu, Bhutan

**Keywords:** Psychology, Human behaviour

## Abstract

We assessed risk/protective factors for cognitive development of Bhutanese children (504 3–5 year-olds, 49% girls, major ethnicities Ngalop 26%, Tshangla 30%, Lhotsampa 34%) using a non-verbal test of cognitive capacity (SON-R) and primary caregiver interviews. Cognitive capacity was related to the family’s SES and whether the family belonged to the primary Buddhist majority ethnic groups (Ngalop or Tshangla) or primarily Hindu minorities (Lhotsampa). In majority families more engagement in Buddhist practices was associated with higher cognitive capacity in children. Minority children were more impacted by parents autonomous-relatedness values. Results demonstrate that cognitive development is dependent on the financial and educational context of the family, societal events, and culture specific risk/protective factors that differ across sub-groups (majority/minority, culture/religion).

## Introduction

It is well known that the cognitive capacity of children (here defined as the adaptive problem-solving skills that are often associated with performance on intelligence tests or other standardized tests) is positively associated with later educational level, salary, and job performance^1,2^. At the same time, this development is not equal for all. Low levels of parental education and low parental incomes (collectively referred to as SES) are associated with low performance on cognitive tests in childhood^3^. This association has been demonstrated both behaviorally and neurologically^4–6^. The same associations are well documented for other related variables such as poverty, focusing on the lower end of the economic spectrum^7–9^ across both high and low income countries^10–12^ extending beyond the family to also include the wealth of the neighborhood in which the families live^13^.

It has also been demonstrated that children whose parents suffer from mental health problems are at risk of delayed cognitive development^14–21^. Effects on child development exist for maternal mental health problems (such as depression and/or anxiety) occurring both during pregnancy^22–26^ and after birth^27,28^. Effects are small, but persistent across income levels, ethnicities, ages, and genders^29^ and can also be measured both behaviorally (in the examples listed above) and neurologically^30,31^.

At the same time, the specific associative pathways linking the context around the child with the cognitive development of that child is subject to cultural variations and should be positioned in the broader societal and cultural context in which children are raised and adult family members live^32–34^. Family income, education, and mental health interact in order to create a socio-economic context for the family that has a collective impact on child development^22–29,35^. To illustrate, recent correlational work suggest that a history of traumatic experiences and current challenges of living in disadvantaged circumstances (financial difficulties, discrimination, loss of socio-economic status) limits the psychological resources available for caregivers to devote to high-quality parenting, resulting in a stronger impact of poor parental mental health on child development outcomes^20,35,36^.

It has been proposed that the impact of multiple stressors on children’s cognitive development are most prominent in low- and middle-income countries due to high poverty rates, lower education levels, and higher prevalence of life stressors^37^. All of these factors might lower caregivers investments in the child’s learning environment, resulting in lower age normed capacities (e.g. children’s cognitive development and intelligence) early in life, and a vicious cycle of low support and poor developmental outcomes that is strengthened over time^38^, see also^39^. Likely, the associations are even more complex since correlations between SES or poverty, mental health of parents, and child development might, could depend on the value systems that exist in a the larger cultural context in which the family lives and the families own cultural beliefs and practices^32,40^.

One central divider between regions in the world, and individuals within these regions, are the religious beliefs which shapes the daily practices of families^41^. Family dynamics, and risk and protective factors that impact the association between poverty, mental health of parents, and child development, might vary depending on the religious beliefs and practices of the family and the religious context in which the family lives. The context surrounding a child might be quite different if raised in a Buddhist, Christian, or secular family and quite different if the family’s religious beliefs coincide with the majority religion in the society in which one live or not. Both SES and religion are part of what defines actual and perceived majority and minority cultures in a society^42^ and the sense of belonging that one has to the community in which one lives^43^. Being engaged in communal activities and participating in social gatherings have been demonstrated to work as a social support buffers that lowers the impact of poor mental health on children’s cognitive development^44^. In addition, religious beliefs are also known to operate as a coping mechanism, being associated with fewer depressive symptoms^45^. In a broader sense, spirituality is also an important component in the creation of meaning, hope, and a sense of relatedness and belonging^46^. With respect to child development, it has been demonstrated that the presence of religious practices among parents and families can be associated with better psychological development of children, including better self-control, social interactions and interpersonal skills^47^. However, the extent to which children’s cognitive development is impacted by religious practices and beliefs is currently not well studied, and seldom studied outside Christianity.

Families also vary in their values towards society and family life, in particular the degree to which they live in a society with individualistic/independent or collectivistic/interdependent values^48,49^, a distinction that also holds for individuals within a particular society. Kagitcibasi^50^ suggested a two-dimensional version of this value system with one axis describing the degree of autonomy and the other the degree of relatedness. Traditional individualistic/independent societies/individuals are considered high on autonomy and low on relatedness in this two-dimensional autonomy-relatedness scale, whereas collectivistic/interdependent societies/individuals are in the opposite quadrant, low on autonomy and high on relatedness. In her work Kagitcibasi^50^ argues that modernization in previously collectivistic/interdependent societies will not lead to individualism/independence but to a new value system defined by high autonomy and high relatedness, incorporating self-determination and agency but with a maintained emotional bond to ones extended family.

In summary, the context in which we are raised impact the cognitive development of children. Factors that have been well documented to impact child development include SES and mental health of parents. In addition, it is possible that religious practices and beliefs (that can create meaning and provide societal buffers against hardship), more general values (that pertain to the relation between the individual and the society in which the individual lives), and the position in society (if a family belongs to a minority group or not) impact these associations, but few studies have assessed these complex relations and their impact on the cognitive development of children.

### Current study

The current study was designed to assess the relation between primary caregiver mental health, SES, and child development in Bhutan, a highly collectivistic/interdependent Buddhist society in the Himalayas^44^. The country includes two larger groups, mostly Buddhist majority Bhutanese (Ngalop, Western Bhutanese of Tibetan origin, and Tshangla, Eastern Bhutanese) and mostly Hindu minority Bhutanese (Lhotsampa, of Nepalese origin). The latter group makes up < 35% of the population, the former groups the overwhelming majority of the remaining percentage^51^. This context has not been studied much in the past. From a developmental psychological perspective only two studies have been published (to our knowledge). Firstly, Astor et al.^39^ compared the impact of maternal depression on infant’s social cognitive abilities more specifically gaze following, for more information about this ability see^52^ in Bhutan and Sweden. Swedish infants’ (*n* = 113) social cognitive ability was related to the primary caregivers’ mental health, with lower abilities in infants of depressed mothers. Bhutanese infants’ (*n* = 105) social cognitive ability were on the same level as their Swedish peers and primary caregivers were, if anything, slightly more depressed than their Swedish counterparts. Despite of this, Bhutanese caregiver’s mental health did not impact their children’s social cognitive ability. Based on this, a cultural mediation model was proposed, arguing that the association between mental health of parents and children’s cognitive development is dependent on the cultural context in which the family lives. Secondly, Juvrud et al.^44^ assessed attention and social perception to faces in the same group of Bhutanese infants. They demonstrated that infant’s attention, but not social perception, was impacted by the mental health of their mothers. The study demonstrated that a strong social context, and frequent social activities, buffered against the negative effect of poor maternal mental health on child development, suggesting that social support can provide a positive impact on child development and limit the impact of poor mental health of primary caregivers on child development. It was proposed that having more people around to support and provide stimulation to children is sufficient to strengthen children’s psychological development. This is consistent with prior findings from a northern European context where a high degree of involvement from the second parent is associated with positive child development already during infancy^53–55^.

The current study assess a different (and larger) group of Bhutanese children on a more standardized, non-verbal, assessment of cognitive capacity (SON-R)^56^. More specifically, 504 families with 3–5-year-old children living in Western Bhutan was invited to the study. The aim is to better understand how SES, mental health of primary caregivers, and the specific religious and cultural context of the family interact with the cognitive capacity of children. In the context of Bhutanese society we choose to assess two aspects of mental health (negative emotional experiences and general mental health) taken from the Third Gross National Happiness Survey^57^, SES, and several aspects of that are central to social life in Bhutan. These variables include religious practices and sense of belonging (as indicators of social support and engagement in community), religious values and ethnicity (as indicators of faith and the socio-cultural context of the family). The last two variables provide an indication of the family’s status in Bhutanese society as the population largely fall into two groups, mostly Buddhist families of Ngalop or Tshangla ethnicity (majority) and mostly Hindu families of Lhotsampa ethnicity (minority). Due to the strong connection between ethnicity and religion we collectively refer to these groups as majority and minority cultures within the Bhutanese context. This is done for two reasons (1) to acknowledge that values, believes, and practices often are difficult to disentangle when comparing homogeneous subgroups with a larger cultural context and (2) that it is might be important to further acknowledge the unique position of Hindu Lhotsampla (which as noted above make up 35% of the population) compared to Buddhist Ngalop or Tshangla (that make up the vast majority of the remaining population) living in a largely Buddhist society.

We further explore how the degree of autonomy and relatedness as a two-dimensional index of societal values^50^ vary over these two groups and how these value systems interact with child development. The goal is to understand how poverty/SES, mental health among caregivers, religion, ethnicity and societal values impact children’s cognitive development in this understudied population.

## Method

### Participants

Five-hundred and four families participated in the study (in accordance with the pre-registration, https://osf.io/yr83j). Out of these, 52 families were excluded as someone other than a primary caregiver brought the children to the test (and where interviewed). These were often fathers that did not identify themselves as a primary caregiver (n = 38) or aunts/uncles/grandparents. An additional 4 families were excluded due to an extreme low birth weight of the child (< 1.5 kg).

**Figure 1:**
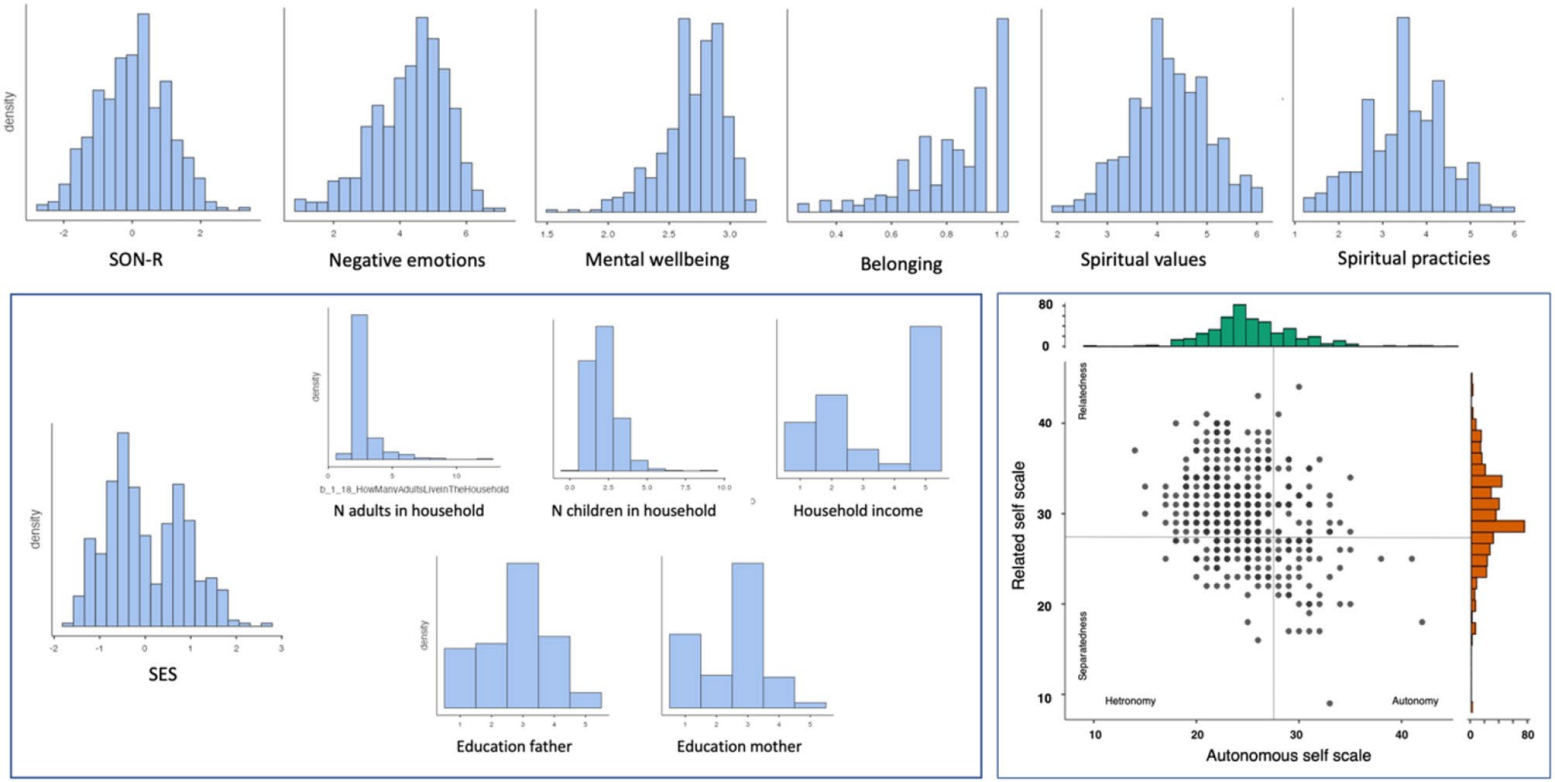
Histograms of variables used in the study (top & left) and a scatterplot of autonomous-relatedness scales, with categorical labels taken from Kagitcibasi^50^.

Primary caregivers included in the study were 393 mothers and 55 fathers (total sample 448 families). They identified as Ngalop (n = 117), Tshangla (n = 136), Lhotsampa (n = 152), other (n = 40), or mixed (n = 3) ethnicity. The vast majority, 99%, of Ngalop and Tshangla identified as being of Buddhist faith. Seventy-four percent of Lhotsampa identified as Hindu, 16% as Buddhist, and 9% as Christian. Only three primary caregivers responded that they were not religious or that they held other beliefs. The average age of mothers and fathers were 31 (*SD* = 4.6 years, range 19–50 years) and 34 years (*SD* = 6 years, range 19–72 years), respectively. The sample of children (48.7% girls) included 3-year-olds (n = 169), 4-year-olds (n = 215), and 5-year-olds (n = 64), with a mean age of 1526 days (*SD* = 231 days, range 1066–2030 days). Figure 1 provides distributions and descriptive statistics for key variables used in this study.

Informed consent was obtained from all participants and their legal guardians prior to the onset of the study (literate parents received both verbal and written information and gave verbal and written consent, illiterate parents were given verbal information and provided verbal concent and signed with their mark, commonly used to sign papers when not being able to write one’s name). A compensation of 500 Ngultrum (approximately 6 €) was provided to each family that participated. The study was approved by the Research Ethics Board of Health, Ministry of Health, Thimphu, Bhutan 2021/048. All research was performed in accordance with relevant guidelines/regulations and in accordance with the Declaration of Helsinki. Consent was obtained from all participants and their legal guardians.

### Procedure

The study was conducted at early child care centers and primary health care centers in the four Westerns regions of Bhutan (Chukha, Paro, Samtse, & Thimphu) from May 16th to July 31st, 2022. Primary caregivers were asked to participate in the study by the professionals working at the data collection site. Parents were given information about the study and asked if they would like to partake in the study. For those that gave informed consent children participated in the SON-R testing which was followed by an interview with the primary caregiver. Two research assistants native to Bhutan conducted the testing (a total of three pairs of assistants collected the data), one assessing the child and the other interviewing the primary caregiver.

### Measures

*SON-R.* Cognitive development of children was measured with SON-R (2–8 years), a non-verbal aptitude test that has successfully been used to assess cognitive development in Australian, Brazilian, Czech, and Slovak children^58^, in addition to children from Germany and the Netherlands^56,59,60^. No cognitive developmental tests have Bhutanese norm data and SON-R was selected due to its non-verbal nature, that it has been used in several countries across the globe, and that it is often used in Northern Europe to assess cognitive development of refugee children that do not speak the language in their current place of residence (Johanna Hellberg, personal communication).

It includes several reasoning (Categories, Analogies, and Situations) and spatial (Mosaics, Puzzles, and Patterns) tasks and it takes approximately 50 min to administer. A clinical psychologist and expert on SON-R (Johanna Hellberg) trained all research assistants on how to administer the SON-R test. The training included two seminars, a series of training videos, practices sessions, and an examination by Johanna Hellberg. The seminars and examination were performed on-line, and the training sessions were conducted in Thimphu, Bhutan. Assistants were not allowed to participate in data collection before being approved by Johanna Hellberg, all of this in agreement with the publisher of SON-R, Hogrefe. The lack of norm-data resulted in us reporting the standardized (z-transformed) average score over the 6 tasks without relying on normalization based on external datasets. As it follows, a value of 1 should be interpreted as a child performing 1 SD above the mean in this group, a value of − 1 should be interpreted as a child performing 1 SD below the average of this group of children.

*Questionnaires*. Primary caregivers were interviewed for approximately 60 min about the family’s life conditions. The interview was structured around, and with the aim to get answers to, a series of questionnaires targeting a larger range of circumstances that those captured in the current study (see Table 1). It was delivered verbally to all participants regardless of whether the adult participants were literate or not, allowing the experimenter to explain concepts and make sure that respondents interpreted the questions in a similar manner. It also resulted in no missing data for any of the questionnaire items. Information about the questionnaires used, including references, are listed in Table 1. The variables used in the current analysis are listed in the following section. The questionnaires are all originally in English and were translated to the language that the primary caregiver was comfortable with and that was spoken by the research assistant conducting the interview. In preparation for this, assistants and project leaders (from Sweden and Bhutan) discussed all questions thoroughly in order to come to a joint understanding of the meaning of each question—in order to allow fluent translations across languages in the actual testing session.

**Table 1:** Questionnaires used in the interview with adult participants.

Topic	Description	N items	Reference
Interview	Info on test situation	5	
Family	Descriptives of family	26	
	Descriptives of child	12	
Poverty	Indicators of poverty	12	^70^*
Autonomy-relatedness scale	Autonomous self	9	^50^
	Relate self	9	
	Autonomous-related self	9	
Early parenting attitudes	Affection and attitude	8	^71^
	Early learning	8	
	Rules and respect	8	
Value of children	Wanting to have children	27	^72^
	Not wanting to have children	21	
Sense of happiness/life satisfaction	Happiness/satisfaction	7	^57^
Mental and emotional wellbeing	Mental well being	12	^57^
	Negative emotional experience	6	
	Positive emotional experiences	5	
	Anxiousness	5	
Religion	Religious practices	5	^57,73^
	Religious values	10	
Sense of belonging and trust	Belonging/trust	5	^57^
Caregivers adverse childhood experiences	Marriage	5	^74^
	Relationship with parents	3	
	Family environment	15	
	Peer/community violence	6	

*Two questions were added to the simple poverty score card: Does the household own any livestock animals? And Does the household produce any cash crops?

### Analysis

The preregistration, https://osf.io/yr83j describe a series of general linear models planned in order to assess the relation between primary caregiver’s mental health (assessed with questionnaires measuring general mental wellbeing and negative emotional experiences) and child development (overall normalized cognitive capacity score from the SON-R test battery). Collinearity statistics (VIF and Tolerance) and Q-Q plots were inspected in order to ensure assumptions were meet. For a detailed list of the questions included to calculate the variables in the above listed analysis (M1-M4 and E1-E2) see Table 2.

**Table 2:** Information about the variables used in the current analysis. In the order they are presented to primary caregivers during the interview.

Description	Response	Questions
Age Ethnicity SES SES SES	Free text 5 categories^1^ 5 categories^2^ Free text Free text	Birthdate of child Ethnicity of caregivers Education level of caregivers Number of adult household members, number of children in the household What is the total household income in Ngultrum (including value of cash crops)
Exclusion criteria	Free text	Birth weight of child
Autonomous self	5 grade Likert scale	1. People who are close to me have little influence on my decisions 2. I do not like a person to interfere with my life even if he/she is very close to me 3 I feel independent of the people who are close to me 4. I lead my life according to the opinions of people to whom I feel close 5. The opinions of those who are close to me influence me on personal issues 6. While making decisions, I consult with those who are close to me 7. On personal issues, I accept the decisions of people to whom I feel very close 8. I usually try to conform to the wishes of those to whom I feel very close 9. I can easily change my decisions according to the wishes of those who are close to me
Relate self	5 grade Likert scale	1. I need the support of persons to whom I feel very close 2. I prefer to keep a certain distance in my close relationships 3. Generally, I keep personal issues to myself 4. The people who are close to me strongly influence my personality 5. I think often of those to whom I feel very close 6. I do not worry about what people think of me even if they are close to me 7. Those who are close to me are my top priority 8. My relationships to those who are close to me make me feel peaceful and secure 9. I do not share personal matters with anyone, even if very close to me
Mental well being	4 grade Likert scale + don’t know option (treated as NaN)	1. Been able to concentrate on what you are doing 2. Lost much sleep over worry 3. Felt you were playing a useful part in things 4. Felt capable of making decisions about things 5. Felt constantly under strain 6. Felt you couldn’t overcome your difficulties 7. Been able to enjoy your normal day-to-day activities 8. Been able to face up to your problems 9. Been feeling unhappy and depressed 10. Been losing confidence in yourself 11. Been thinking of yourself as a worthless person 12. Been feeling reasonably happy, all things considered
Negative emotional experience	7 grade Likert scale	During the past four weeks, how often have you felt the following moods/emotions? 1. Anger 2. Selfishness 3. Jealousy 4. Fear 5. Worry 6. Sadness
Religious practices	6 grade Likert scale	1. Offering food to the monks/nuns or making merit to homeless 2. Praying 3. Listening to the sermons or reading or viewing the Dharma activities/attending churches 4. Practicing basic religious beliefs (doctrine^a^) 5. Practicing meditation
Religious values	6 grade Likert scale	1. Expressing gratitude to one’s parents 2. Re-paying people who provided assistance 3. Accepting guilt 4. Forgiving 5. Practicing principles of sufficiency economy^b^ 6. Helping the needy 7. Providing opportunity to the others 8. Saving lives 9. Visits local temples/churches and other places of spiritual significance within the community 10. Consider cause and effect relationship (Karma) in the course of daily life
Sense of belonging	3 Grade Likert scale + don’t know option (treated as NaN)	1. How would you describe your sense of belonging to your local community 2. Would you say this is a neighborhood where neighbors help one another out 3. In the last month how often did you socialize with your neighbors

^1^Ethnicity = Ngalop, Tshangle, Lhotshampa, Other, Mixed ethnicity. ^2^Education = No formal schooling, completed primary school, completed, high school, completed collage, other.

More specifically, the pre-registration lists a series of linear models where Model 1 include gender (boy = 1, girl = 2) and age of the child (a categorical variable with age in years was listed in the pre-registration but here a continuous age variable, in days, is included instead) the two measures of mental health as independent variables and child development as the dependent variable. Following this, a series of increasingly complex models were assessed, each included the variables with a *p*-value below 0.1 from the prior analysis while adding additional variables (note that the results remain identical if only significant variables would have been moved on to the next analysis). Model 2 included socioeconomic status mean (z(yearly family income/number of household members), z(mean(educational level mother, educational level father))), religious practices (describing traditional religious practices in Bhutan and thus naturally associated with Buddhism), religious values (reflecting Buddhist values), and the families sense of belonging (to the local community in which they live). Model 3 included ethnicity, a categorical variable with many diverse ethnic groups were included in the pre-registration but here a categorical variable with two levels (minority ethnic group = Lhotsampa and majority ethnic groups = Ngalop and Tshangla) is included instead. Model 4 included interaction effects for all remaining variables.

In addition to the pre-registered analysis the role of ethnicity was assessed further by including a final stage with separate analysis for majority culture families of Ngalop (n = 117) and Tshangla (n = 136) ethnicity that to 99% identify as Buddhists in this sample (E1) and minority families of Lhotsampa (n = 152) ethnicity that to 74% identify as Hindu in this sample (E2). This analysis included the variables noted above, and in addition the two-dimensional autonomous/relatedness scale described in the introduction. More specifically, the variables autonomous self (the degree to which the adult relies on close others for financial support and important decisions in life) and related self (the degree to which the adult relates to close others for social and emotional support or if they see themselves as strong on their own)^50^. These are included in order to cover broader spectrums of cultural values that are associated with, but distinct from, ethnicity/religion/SES.

## Results

Table 3 includes descriptive statistics of all continuous variables included in the analysis. Cronbach’s alpha is good (> 0.8) for central variables such as SON-R, caregiver’s negative emotional experiences, primary caregiver general mental wellbeing, and religious values. Religious practices and sense of belonging assess different situations that might create an overall perception of activities and situations, without the assumption that there is a single underlying latent structure, and Cronbach’s alpha is lower for these measures (0.6-0.7). A correlation table with all variables used in the entire results section can be found in Table 4.

**Table 3:** Depict descriptive statistics of central variables.

	SON-R	Age	SES	PCNE	GMW	Practices	Values	Belonging	Autonomous	Relatedness
N	448	448	425	448	448	448	448	448	448	448
Mean	0	1526	0.0063	4.4	2.71	3.53	4.25	0.817	24.7	29.8
SD	1	231	0.33	1.11	0.265	0.892	0.785	0.168	4.01	4.88
Cronbach’s alpha	0.891	–	–	0.802	0.818	0.625	0.838	0.675	0.608	0.54

PCNE = Primary Caregiver Negative Emotions, GMW = General Mental Wellbeing, Practices/Values = Religious Practices/Values, Autonumous/Related = Autonumous and Related self.

**Table 4:** Correlation matrix with all variables used in pre-registered and exploratory analysis.

		SON-R	Age	SES	PCNE	GMW	Practices	Values	Belonging	Autonomous	Relatedness	Gender*
SON-R	rxy	–										
	*p*	–										
Age	rxy	***0.640***	–									
	*p*	** < *.001***	–									
SES	rxy	***0.203***	− 0.089	–								
	*p*	** < *.001***	0.066	–								
PCNE	rxy	0.035	***0.188***	− ***0.198***	–							
	*p*	0.466	** < *.001***	** < *.001***	–							
GMW	rxy	0.015	***0.105***	− ***0.153***	***0.141***	–						
	*p*	0.747	***0.026***	***0.002***	***0.003***	–						
Practices	rxy	***0.114***	0.020	***0.118***	− 0.030	− 0.011	–					
	*p*	***0.016***	0.677	***0.015***	0.523	0.816	–					
Values	rxy	***0.101***	− 0.016	***0.150***	0.049	0.004	***0.524***	–				
	*p*	***0.033***	0.742	***0.002***	0.298	0.925	** < *.001***	–				
Belonging	rxy	− ***0.008***	***0.113***	− ***0.199***	***0.145***	***0.123***	0.020	0.008	–			
	*p*	***0.873***	***0.017***	** < *.001***	***0.002***	***0.009***	0.671	0.866	–			
Autonomous	rxy	− 0.089	− 0.080	0.069	0.063	− ***0.119***	− 0.059	− 0.017	− 0.002	–		
	*p*	0.059	0.089	0.157	0.183	***0.012***	0.209	0.724	0.974	–		
Relatedness	rxy	− 0.063	0.082	− ***0.123***	− ***0.098***	***0.124***	− 0.078	− 0.070	***0.095***	− ***0.325***	–	
	*p*	< .181	0.083	***0.011***	***0.039***	***0.009***	0.100	0.139	***0.044***	** < *.001***	–	
Gender*	rxy	0.060	0.004	0.025	0.036	− ***0.104***	0.031	0.080	0.050	0.083	− 0.049	–
	*p*	0.209	0.939	0.613	0.450	***0.028***	0.511	0.093	0.295	0.078	0.302	–

PCNE = Primary Caregiver Negative Emotions, GMW = General Mental Wellbeing, Practices/Values = Religious Practices/Values, Autonomous/Related = Autonomous and Related self. All Pearson correlations except * = Spearman. Significant correlations noted with bold and italic.

The first step of the analysis included SON-R as dependent variable and children’s gender and age, as well as caregivers’ general mental wellbeing, and negative emotional experiences as independent variables. Model 1 demonstrated that children’s age (older children have higher SON-R scores) and caregiver’s negative emotional experiences (more negative experiences associated with lower SON-R scores) explained a significant amount of variance in SON-R performance, *F*(4,442) = 80.4, *p* < 0.001, R^2^ = 0.421, R^2^_adj._ = 0.416 (for analysis M1–M3 see Table 5).

**Table 5:** Pre-registered models assessing the relation between children’s cognitive development (SON-R) and characteristics of children and their primary caregivers.

Model	Predictor	Estimate	SE	t	*p*
1	Intercept	− 3.7993	0.4573	− 8.308	** < *.001***
	Child’s age	0.0029	1.60e−4	17.86	** < *.001***
	Child’s gender	0.1197	0.1107	1.08	0.280
	Primary caregiver negative emotional experiences	− 0.0777	0.0337	− 2.31	**0.021**
	Primary caregiver general mental wellbeing	− 0.1312	0.1402	− 0.936	0.350
2	Intercept	− 4.5476	0.3351	− 13.571	** < *0.001***
	Children’s age	0.0029	1.51e−4	19.126	** < *.001***
	Primary caregiver negative emotional experiences	− 0.0232	0.0320	− 0.725	0.469
	Family’s SES	0.2796	0.0430	6.502	** < *.001***
	Primary caregiver’s religious practices	0.0574	0.0446	1.287	0.1999
	Primary caregiver’s religious values	0.0584	0.0511	1.142	0.254
	Primary caregiver’s sense of belonging	− 0.2297	0.2083	− 1.103	0.271
3	Intercept	− 4.8347	0.2685	− 18.00	** < *.001***
	Children’s age	0.0029	1.52e−4	18.86	** < *.001***
	Family’s SES	0.249	0.0437	5.70	** < *.001***
	Primary caregiver’s ethnicity	0.2853	0.0747	3.82	** < *.001***

Model 2 included the significant predictors from Model 1 (children’s age and caregiver’s negative emotional experiences) along with the family’s SES, the primary caregiver’s religious practices, religious values, and sense of belonging as independent variables and SON-R as the dependent variable. The model demonstrated that the primary caregivers’ negative emotional experiences no longer explained a significant amount of variance in SON-R performance. Instead children’s age and the families’ SES (higher SES is associated with higher SON-R scores) explained a significant amount of SON-R variance, *F*(6,418) = 69.2,* p* < 0.001, R^2^ = 0.498, R^2^_adj_ = 0.491.

Model 3 included significant predictors from Model 2 (children’s age and family’s SES) and ethnicity of the primary caregiver as independent variables and SON-R as the dependent variable. The model demonstrated that all independent variables explained a significant amount of variance in SON-R performance, with lower SON-R for minority children of Lhotsampa origin (mean score − 0.272, CI_95_ = − 0.427 to − 0.118) than majority children from Ngalop or Tshangla origin (mean score 0.080, CI_95_ = − 0.040–0.201), *F*(1,321) = 12.6, *p* < 0.001. According to Model 4, no significant interactions (2- or 3-way) between independent variables from Model 3 and the dependent variable (SON-R) could be observed. The model remained significant (*F*(7,374) = 55.7, *p* < 0.001, R^2^ = 0.510, R^2^_adj._ = 0.501).

### Exploratory analysis

The sequential addition of blocks of variables in this series of analysis is described in the pre-registration and we see this as an important way to understand what happens in this dataset and in interactions between variables (for example the fact that primary caregiver mental health disappears as a significant variable when adding SES is hard to see in a single model with all variables). However, for sake of transparency, when all variables are added in a single multiple regression analysis the results from Model 3 hold (*F*(9,371) = 44.5, *p* < 0.001, R^2^ = 0.519, R^2^_adj_ = 0.507) with significant contributions from children’s age (*p* < 0.001), SES (*p* < 0.001), and minority status (*p* < 0.001) suggesting that the results are robust to variations in analytic choices.

Table 6 report differences between minority and majority children and their families for all variables used in Model E1 and E2 below. Note that two additional variables are included in these analysis (above what was specified in the pre-registration), focusing on autonomous and related self as a two-dimensional indicator of collectivistic/interdependent and individualistic/independent values^50^. The analysis demonstrated that the two groups of families differ on several dimensions, in particular their SES (lower SES in minority Lhotsampa), their religious values (majority Buddhist primary caregivers report agreeing with traditional Buddhist values to a larger degree), and their sense of belonging to the local community (larger in minority Lhotsampa). Table 3 illustrate that Cronbach’s alpha is questionable (0.61 and 0.54) for these variables, something that needs to be taken into account when reflecting on the results below.

**Table 6:** Report independent t-tests for all variables used in Model 5 separating the sample by Lhotsampa and Ngalop/Tshangla ethnicities.

Factor					Minority	Majority
	t-value	df	*p*-value	Cohen’sd	Lhotsampa (mean/*SD*)	Ngalop/Tshangla (mean, SD)
SON-R	− 3.54	403	** < *0.01***	− 0.36	− 0.27/0.96	0.08/0.97
SES	− 4.86	380	** < *0.01***	− 0.52	− 0.27/0.82	0.14/0.79
Children’s age	1.38	403	0.17	0.14	1537/*221*	1505/*235*
Primary caregiver’s autonomous self	− 0.15	403	0.88	− 0.02	24.57/*4.40*	24.61/*3.76*
Primary caregiver’s related self	0.35	403	0.72	0.036	29.86/*5.03*	29.68/*4.84*
Primary caregiver’s religious practices	− 0.58	403	0.56	− 0.06	3.51/*0.85*	3.56/*0.88*
Primary caregiver’s religious values	− 2.33	403	***0.02***	− 0.24	4.15/*0.73*	4.34/*0.78*
Primary caregiver’s sense of belonging	3.12	403	** < *0.01***	0.32	0.85/*0.16*	0.80/*0.17*

Significant *p*-values (*p* < 0.05) marked with bold and italic.

The model (E1) for majority Bhutanese (Ngalop & Tshangla) demonstrated that children’s age, family’s SES, related self (low relatedness is associated with higher SON-R scores), and religious practices (more engagement in religious practices often associated with Buddhism is associated with higher SON-R scores) explained a significant amount of variance in SON-R performance (*F*(7,237) = *p* < 0.001, R^2^ = 0.545, R^2^_adj_ = 0.532). For minority Bhutanese (Lhotsampa) the model demonstrated that children’s age, family’s SES, and the degree of autonomous self (low autonomy is associated with higher SON-R scores), and related self (low relatedness is associated with higher SON-R scores) contributed to children’s performance on SON-R, see Table 7 (*F*(7,129) = *p* < 0.001, R^2^ = 0.511, R^2^_adj_ = 0.484).

**Table 7:** Separate analysis of SON-R performance for majority (Ngalop & Tshangla) and minority (Lhotsampa) Bhutanese ethnicities.

Culture	Predictor	Estimate	SE	t	*p*
Ngalop & Tshangla	Intercept	− 4.38914	0.6636	− 6.614	** < *.001***
	SES	0.18548	0.0549	3.376	** < *.001***
	Children’s age	0.00308	1.92e−4	16.016	** < *.001***
	Primary caregiver’s autonomous self	0.00433	0.0129	0.335	0.738
	Primary caregiver’s related self	− 0.01971	0.0097	− 2.042	***0.042***
	Primary caregiver’s religious practices	0.15841	0.0583	2.717	***0.007***
	Primary caregiver’s religious values	− 0.02773	0.0635	− 0.437	0.663
	Primary caregiver’s sense of belonging	− 0.25588	0.2714	− 0.943	0.347
Lhotsampa	Intercept	− 2.70364	0.8217	− 3.290	** < *.001***
	SES	0.23694	0.0763	3.105	***0.002***
	Children’s age	0.00288	2.71e−4	10.654	** < *.001***
	Primary caregiver’s autonomous self	− 0.03564	0.0144	− 2.482	***0.014***
	Primary caregiver’s related self	− 0.03727	0.0127	− 2.931	***0.004***
	Primary caregiver’s religious practices	− 0.04941	0.0830	− 0.595	0.553
	Primary caregiver’s religious values	0.13709	0.0987	1.388	0.167
	Primary caregiver’s sense of belonging	− 0.08814	0.3862	− 0.228	0.820

Significant *p*-values (*p* < 0.05) marked with bold and italic.

## Discussion

Bhutanese caregivers with a low SES background are more likely to suffer from poor mental health and have children with a lower level of cognitive development, compared to peers that live in high-SES households. One likely explanation for this is that being poor and having a low educational background limits opportunities (material and mental) and increases the risk of an impoverished environment for the child to grow up in, with direct consequences for cognitive development of children^5,61^. Poor mental health is also associated with poverty^62^, but may not, in the current context, assert a primary direct impact on child development.

The fact that primary caregiver’s mental health did not impact child development in the final models, that also include SES, can perhaps be attributed to the collectivistic properties of the Bhutanese society, defined by a high degree of relatedness and low autonomy (a description that nicely capture the current population, see Fig. 1). In this context there are many people being close to the parent–child dyad, people that can provide a buffer against a direct association between mental health and child development—others can step in and provide stimulation, support, and care that fill some of the social and emotional gap that risk being present when a caregiver suffers from poor mental health. This buffering would protect against low quality (and quantity) social interactions more than material challenges associated with poverty and/or lack of educational. After all, a poor family does not obtain (proportionally speaking) more resource if there are more caring adults in the household. But these adults can, in the best of worlds, provide support and provide enrichment, and rewarding social contacts, that the children need to thrive. According to this line of logic, children growing up in highly individualistic societies are to some extent more vulnerable to caregiver mental health problems, as there are fewer significant others that can support and buffer during daytime (in those cases where there is a second caregiver that is working during daytime). Perhaps the association between caregiver’s mental health and child development are strongest in such societies, a vulnerability particularly prominent in WEIRD (Western, Educated, Industrialized, Rich, Democratic; Henrich et al.^63^) societies in the global North. At the same time, it is important to note that the positive effect attributed to Bhutanese society in this line of argument, with respect to social support, is a supposition based on cultural values that pertain to the entire sample (the high degree of relatedness and low autonomy depicted in the lower right panel of Fig. 1 and descriptive data from Table 3). Further studies need to directly assess individual differences in social support in order to further test the validity of this suggestion, something that has been done in prior work looking at the cognitive development of infants^44^. In line with these sets of arguments, it is possible that poverty and mental health of primary caregiver’s impact child development differentially across cultures. In the currently assessed context it appears that poverty is a more profound risk factor for child development than poor mental health. In a different context the relative impact of these variables might be quite different.

The study provides an illustration of the importance of considering more than country of origin in these types of analysis. Separate analysis for majority Buddhist and minority Hindus illustrates that the cultural contexts that the families live in has a strong impact on the factors that impact children’s cognitive development. In majority culture families, the caregiver’s religious practices turn out to be an important determinant of children’s cognitive capacities. In this context it is important to note that Buddhism is a very central cornerstone of Bhutanese society, and most Buddhists make regular visits to monasteries, participate in religious ceremonies/holidays. Overall, Buddhism is a well-integrated component of social life^57^. With this in mind, it is possible to view religious practices as an indication of societal involvement and an indicator of the social network of families. More specifically, we argue that families who participate in many societal functions and religious communal practices have a stronger societal connection and this provides benefits for child development, perhaps both through the extended network and more enrichment for children. It is also possible that religious practices are highly important for this group because of the association between this variable the parents religious and/or spiritual beliefs which might strengthen parents in their daily struggles. Table 6 suggest that the strength of religious values differ between majority and minority families. At the same time, the models included religious values and no direct or indirect effects were observed. We also see that primary caregivers with a lower degree of relatedness (more focus on the close family as opposed to the larger family network) have children with more positive child outcomes. Perhaps a sign of increased investment and time devoted to the child.

Contrary to this, minority Hindu children live in families that on average have lower SES (Table 3), they are impacted by other, secular, family values. In this community, it appears beneficial to the child if primary caregivers are less autonomous and have lower levels of relatedness than other Hindu caregivers (the latter effect of relatedness is also evident in majority cultures as noted above). This combined pattern of cultural values (low autonomy, low relatedness) is not well described in the literature^50^ and is, in this context unexpected. The exact nature of this relation is beyond the scope of the current data but some speculations are possible as follows.

We see two related differences between these groups that were not explored in the current study. First of all, Hindu primary caregivers (often mothers) are frequently married into a family and might not have established close bonds with their new family yet, while still being highly dependent on that family for their daily life, particularly under poverty (Table 3). This is, by itself, a vulnerable situation to be in, and not a common practice in the majority Buddhist context. Secondly, in a Bhutanese context, inheritance follows matrilineal family in western, central and some parts of eastern Bhutan (dominated by Ngalop/Tshangla ethnic groups with Buddhist believes) and as a result more than 60% of rural women inherit land and have land registered in their names and 45% of urban women have properties registered in their names^64^. The prevalence of ever having experienced intimate partner violence in districts that practices matrimonial inheritance ranges from 22% in Zhemgang to 49% in Punakha. However, in the Southern (Lhotsampha, Hindu dominant) parts of Bhutan, where patrilineal inheritance norm is practiced, the prevalence of ever experienced interpersonal violence is very high ranging from 58% in Samtse to 70% in Tsirang^65^. Based on these numbers it is possible that being a woman and mother in a Hindu Bhutanese context is quite different from the experiences of their Buddhist counterparts in Bhutan, with more dependence on others and less emotional attachment to the family in which one lives. We know from other contexts that vulnerability sometimes lead primary caregivers to focus their attention on the child, with more warmth and overprotection than what can be observed in caregivers that do not suffer from such hardships^66,67^. For a young child it is possible that this results in a temporary boost in cognitive development, with in this group, derived from the positive interaction and sustained focus of the primary caregiver (mother). Again, this is only a speculation and a suggestion for how to interpret the findings that we see. There is a sufficiently large literature pointing to a negative long-term effect of parental vulnerability on child development (for example, Tu et al.^20^) to assume that this effect will not result in a long-lasting cognitive boost for children living in hardship. In the end, the reason for why this set of values are beneficial to the child is unclear and requires more research.

What we can demonstrate with certainty, at this point, is that cultural/religious values differ across groups (even within a country) and that these sets of values impact the children as they grow up in different ways. Culture is made up of complex system of contextual factors, social and spiritual values, and believes that jointly impact the context in which the child is brought up and the development of children. These factors differ across ethnic, religious, and socio-cultural groups. These findings support the cultural mitigation hypothesis and at the same time provides a clear instantiation of Bronfenbrenner’s bioecological model^32,40^ by demonstrating that multiple cultural layers impact child development, both society as a whole, the families religion, and the family’s position in society (majority/minority status) all matters for child development. Similar results have previously been demonstrated when comparing ostracism in interdependent farmers and independent herder communities^68^ suggesting that economic activities of social groups within a culture impact the psychological development of children early in life.

Before concluding, three aspects of the results need to be discussed in more detail. Firstly, with respect to caregiver mental health, results are related to the adults’ negative emotional experiences and not their general mental health. This is perhaps not surprising (though not specified in the pre-registration) given that the general mental health questions relate to the mental life of participants in the broadest possible sense including sleep, confidence, and the capacity to make decisions, as well as questions more directly related to depression and anxiety. The negative emotional experiences on the other hand relate directly to the extent to which the participants had experienced anger, selfishness, jealousy, fear, worry, and sadness in the last two weeks. This is a more straightforward way to ask about feelings closely connected to depression and anxiety, the very mental health problems that have been associated with poor child development in past studies in other contexts^20,22,29,39,69^. The reasonably good Cronbach’s alpha for both measures (and SON-R) suggests that reported differences might not be attributed to different reliability across measures.

Secondly, there are some scales where Cronbach’s alpha is smaller than what would have been preferred. Religious practices and primary caregiver’s sense of belonging to the community in which they live are two measures with lower reliability. As discussed above, it is perhaps unreasonable to expect, or even want, a high alpha for these measures as the different questions that are used to construct these variables assess different behaviors that caregivers may or may not engage in or experience. The questions map out different contexts in order to get a broad assessment of different behavioral patterns that, when put together, can inform us about the lives of caregivers. This is something very different from the multiple questions assessing, for example, mental health or the different facets of the SON-R test that jointly speak to the cognitive developmental level of the child. At the same time, the low Cronbach’s alpha for autonomous and related self is more challenging to understand. Here a large array of very similar questions are asked to participants, and a higher reliability was expected. Currently, the reason behind the relatively low Cronbach’s alpha for these variables is not known.

Third, as this is a cross-sectional study, we can only report on the concurrent association between the primary caregiver’s responses and children’s performance on the SON-R test. We discuss and contextualize these findings in relation to children’s developmental trajectory and the larger socio-cultural context of the family. It is in this regard important to emphasize that developmental statements are interpretations and that they would need to be followed up with new longitudinal samples that assess changes in context, values, and opportunities of families and relate this to change in cognitive development of children.

### Summary

In sum, the study demonstrates that Bhutanese children’s cognitive development is highly dependent on SES and to a much smaller degree on primary caregiver’s mental health than what has been reported from other contexts. Additional risk/protective factors exist and these are expressed differently depending on the cultural and religious context that the family and the child lives in. It is quite a different thing to be raised as a part of a Hindu minority or a Buddhist majority context in Bhutan and each of these contexts come with their own risk/protective factors that impact child development in unique ways.

## Data Availability

The datasets generated and/or analyzed during the current study are available in the OSF repository, https://osf.io/yr83j/?view_only=d96a4a06c16d4dcb89eb1117c971a04c.
